# Lymphocyte count as a sign of immunoparalysis and its correlation with nutritional status in pediatric intensive care patients with sepsis: A pilot study

**DOI:** 10.6061/clinics/2016(11)05

**Published:** 2016-11

**Authors:** Talita Freitas Manzoli, Artur Figueiredo Delgado, Eduardo Juan Troster, Werther Brunow de Carvalho, Ana Caroline Barreto Antunes, Desirée Mayara Marques, Patrícia Zamberlan

**Affiliations:** I Hospital das Clínicas da Faculdade de Medicina da Universidade de São Paulo, Pediatria, São Paulo/SP, Brazil; II Hospital Cruz Azul, São Paulo/SP, Brazil

**Keywords:** Immunoparalysis, Lymphopenia, Nutrition, Sepsis, Septic Shock, Nutritional Status

## Abstract

**OBJECTIVES:**

Developing malnutrition during hospitalization is well recognized worldwide, and children are at a relatively higher risk for malnutrition than adults. Malnutrition can lead to immune dysfunction, which is associated with a higher mortality rate due to sepsis, the most frequent cause of death in pediatric intensive care units (PICUs). The aim of this study was to investigate whether malnourished patients are more likely to have relative or absolute lymphopenia and, consequently, worse prognoses.

**METHODS:**

We enrolled 14 consecutive patients with sepsis whose legal representatives provided written informed consent. Patients were classified as normal or malnourished based on anthropometric measurements. As an additional evaluation of nutritional status, serum albumin and zinc were measured on the 1^st^ and 7^th^ days of hospitalization. Lymphocyte count was also measured on the 1^st^ and 7^th^ days. Clinicaltrials.gov: NCT02698683.

**RESULTS:**

Malnutrition prevalence rates were 33.3% and 42.8% based on weight and height, respectively. Laboratory analyses revealed a reduction of serum albumin in 100% of patients and reduction of zinc in 93.3% of patients. A total of 35% of patients had fewer than 500 lymphocytes/mm^3^ on their first day in the PICU. Lymphocyte counts and zinc concentrations significantly increased during hospitalization.

**CONCLUSIONS:**

Nutritional evaluations, including anthropometric measurements, were not correlated with lymphocyte counts. Lymphocyte counts concomitantly increased with zinc levels, suggesting that micronutrient supplementation benefits patients with sepsis.

## INTRODUCTION

The adequate delivery of nutrients is essential for maintaining metabolism and body composition among critically ill patients. Studies conducted in Brazil and England have estimated that 50% of patients in pediatric intensive care units (PICUs) experience malnutrition during hospitalization 1,2. Compared with adults, children are more likely to experience nutritional deficiencies because they have higher metabolic rates and caloric needs that promote growth and development 3,4.

Sepsis is the most frequent cause of death in the PICU, accounting for approximately 50% of all deaths 5. The adequate intake of nutrients is essential for organs and systems to function properly; this includes the immune system, whose performance is critical to effectively protect the body against pathogens 6,7.

Inflammatory proteins, zinc, selenium, vitamin C, vitamin A and vitamin E are key players in the immune response 6,8,9,10,11,12. These nutrients play a key role in the innate immune response by regulating free radical production and participating in the development of cells, such as neutrophils and natural killer cells, as well as the adaptive immune response by participating in lymphocyte maturation and activity.

As defined by the Adult and Pediatric Consensus Guidelines of 1992 and 2005 13,14, catabolism predominates over anabolism during sepsis, thereby leading to an increase in protein intake and, often, a negative nitrogen balance. This inflammatory response is associated with organ dysfunction and can cause additional nutritional deterioration 1,2,15.

Sepsis also produces an intense release of pro-inflammatory mediators (SIRS) followed by a compensatory anti-inflammatory phase (CARS) 16. The proper functioning of the immune system depends on the appropriate balance between pro- and anti-inflammatory responses.

Immunoparalysis is a clinical syndrome characterized by an inability to restore homeostasis between pro- and anti-inflammatory systems and the persistence of a severe anti-inflammatory state 16,17,18,19,20,21,22,23. This state is primarily detected via stable or diminished monocyte human leukocyte antigen-DR (HLA-DR) levels after the peak of the anti-inflammatory response 17,24,25,26,27 and some authors have suggested that it is signaled by prolonged lymphopenia 28,29,30.

A previous study by our group provided one example of the effect of this syndrome in which patients with immunoparalysis showed a mortality rate of 46%, whereas patients without immunoparalysis showed a mortality rate of 7% 27. Other studies have corroborated these results 19,20,21,25,31.

Since nutritional deficiencies can lead to immune system dysfunction 7,32, we infer that malnourished patients are more likely to suffer from immunoparalysis. Thus, interventional studies have been performed using nutrient supplementation to reverse this condition </emph>^32^,^33^.

In the present study, we assessed whether children and adolescents admitted to a tertiary PICU showed alterations in total lymphocyte count. Moreover, we evaluated the correlation between lymphocyte count and nutritional status. To the best of our knowledge, this study is the first to investigate this relationship. We hypothesized that malnourished patients would show relative or absolute lymphopenia and, therefore, a potentially worse prognosis.

## METHODS

We performed a prospective cohort pilot study of patients with sepsis (as defined by the 2005 conference) 13 who were admitted to a level I PICU. We evaluated their nutritional profile and lymphocyte count.

This study was approved by the Ethics Committee of Hospital das Clínicas, FMUSP. Patients’ legal guardians were provided with informed consent documents and freely chose whether to participate in this study.

We included all patients admitted to the PICU during the study period who were diagnosed with severe sepsis or septic shock. Patients were not included if they died within 24 hours of PICU admission or if their legal guardians did not provide informed consent.

Patients were followed until death or discharge from the ICU. To define patient nutritional profiles, we performed classic anthropometric measurements at ICU admission as well as tests of their albumin, C-reactive protein and serum zinc levels during the first and seventh days of hospitalization.

The anthropometric measurements included length, weight, triceps skinfold thickness (TSF; a measurement of body fat) and mid-arm muscle circumference (MAMC; a measure of protein mass). These values were compared with those considered normal for the patient’s age group according to the World Health Organization classification. MAMC was obtained using the following formula: MAMC = AC – (p x TSF) (mm), where AC = arm circumference in mm and p = 3.1416.

Patients were classified as either well-nourished or malnourished using z-scores based on the 2006 definitions of the World Health Organization.

Hepatic protein production was assessed indirectly by measuring the visceral protein albumin levels of the patients. Furthermore, we measured the concentration of inflammatory C-reactive protein, which is also produced hepatically. The concentration of C-reactive protein was determined by nephelometry.

Albumin concentrations were measured by reaction with bromocresol green in acid medium.

Atomic absorption spectrometry was performed to quantify the zinc content in plasma. Blood samples were collected in EDTA metal-free tubes. The samples were hemolyzed and discharged. The reference values ranged from 0.50 to 1.10 µg/mL.

We evaluated patient immune systems by measuring the total lymphocyte counts as well as the CD4+ and CD8+ cell counts using flow cytometry.

Variables were analyzed using the Mann-Whitney U test and Pearson’s correlation.

## RESULTS

We evaluated 14 patients (10 females), two of whom died before PICU discharge. Sepsis arose from lung, meningeal, gastrointestinal tract or skin sources across the sample.

The median age of the patients was 24 months; the median body mass index z-score (zBMI) was -1.2; and the median height for age z-score (zH/A) was -1.64. Approximately 50% of the children were classified as malnourished on the first day of hospitalization according to their weight for age (W/A). Approximately 66.6% of the sample could be classified as malnourished according to median upper arm circumference (MUAC), and 53% of children were classified as having low H/A on admission.

**Figure 1:**
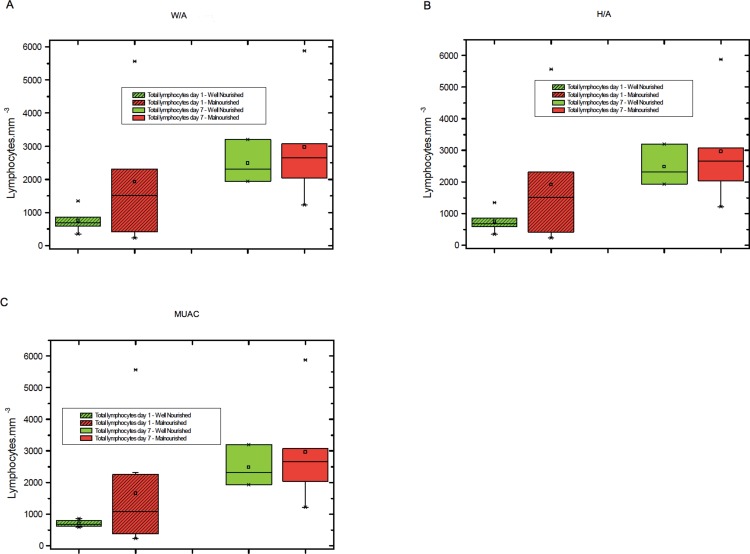
A) Total lymphocyte counts on days 1 and 7 among malnourished and well-nourished children according to weight for age (W/A), B) Total lymphocyte counts on days 1 and 7 in malnourished and well-nourished children according to height for age (H/A), C) Total lymphocyte counts on days 1 and 7 in malnourished and well-nourished children according to median upper arm circumference (MUAC).

**Figure 2:**
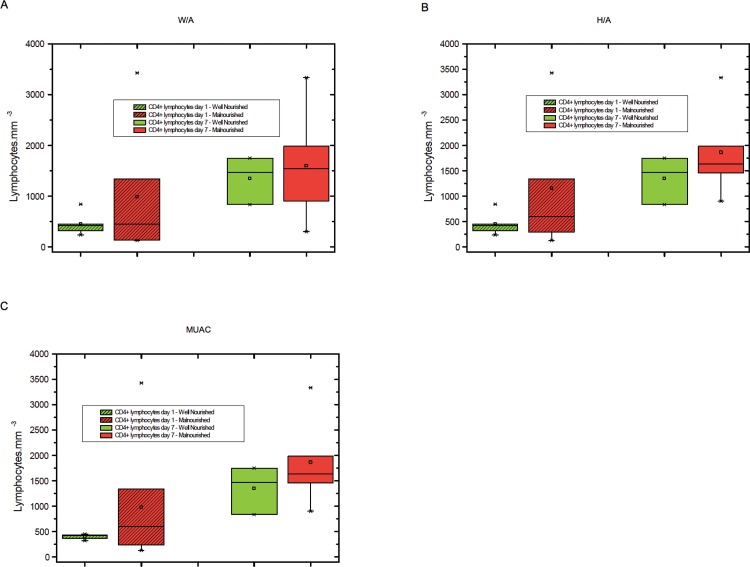
A) CD4+ lymphocyte counts on days 1 and 7 in malnourished and well-nourished children according to weight for age (W/A), B) CD4+ lymphocyte counts on days 1 and 7 in malnourished and well-nourished children according to height for age (H/A), C) CD4+ lymphocyte counts at days 1 and 7 in malnourished and well-nourished children according to median upper arm circumference (MUAC).

**Figure 3:**
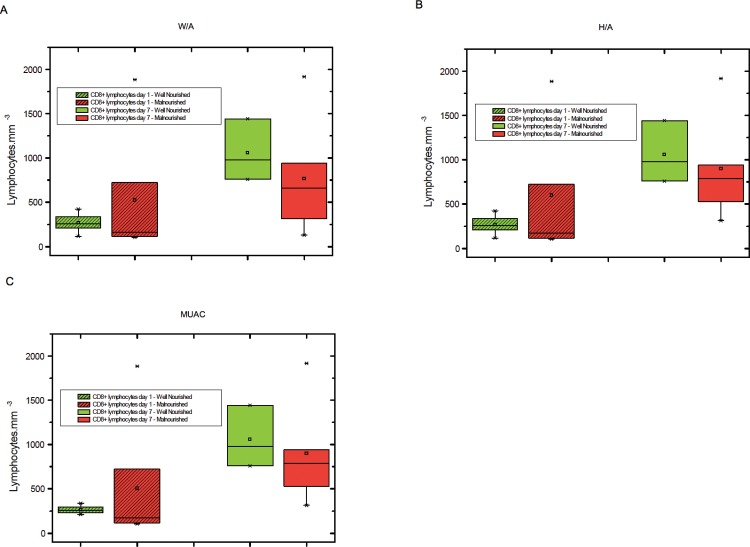
A) CD8+lymphocyte counts on days 1 and 7 in malnourished and well-nourished children according to weight for age (W/A), B) CD8+ lymphocyte counts on days 1 and 7 in malnourished and well-nourished children according to height for age (H/A), C) CD8+ lymphocyte counts on days 1 and 7 in malnourished and well-nourished children according to median upper arm circumference (MUAC).

The levels of total lymphocytes, CD4+ lymphocytes and CD8+ lymphocytes on days 1 and 7 were not significantly different between malnourished and well-nourished children (Figures 1-3).

All of the patients showed albumin concentrations below the reference value upon admission, and 77% maintained hypoalbuminemia on the 7^th^ day of hospitalization. The median hemoglobin level upon admission was 9.7 g/dL, and 22% of the patients had levels below 7 g/dL; Moreover, 93.3% of patients showed low concentrations of zinc on the first day of hospitalization, and 27% maintained those levels on the 7^th^ day of hospitalization. A significant increase in zinc concentration was observed between the 1^st^ and 7^th^ days of hospitalization (*p*=0.0013).

We also observed that 35% of patients had severe lymphopenia (less than 500 lymphocytes) upon admission to the ICU.

**Figure 4:**
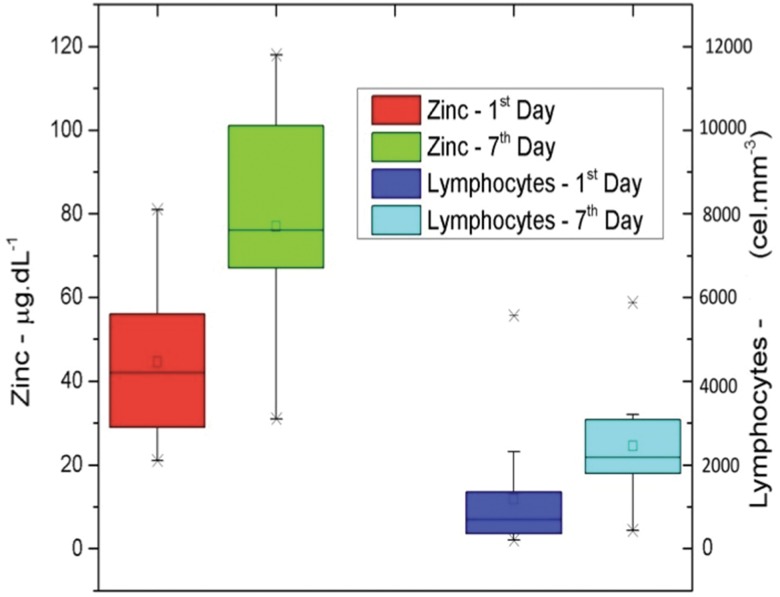
Increase in zinc dosage and lymphocyte count between the first and seventh days. A positive Pearson's correlation was found between zinc levels and lymphocyte counts. The Pearson's correlation coefficients between the CD4+ lymphocyte counts and zinc levels on the first day of hospitalization ranged from -0.6618 to 0.4167. The correlation between the CD4+ lymphocyte counts and zinc levels on the seventh day of hospitalization ranged from -0.5659 to 0.7438. The correlation between the CD8+ lymphocyte counts and zinc levels on the first day of hospitalization ranged from -0.6668 to 0.4092. The correlation between the CD8+ lymphocyte counts and zinc levels on the seventh day of hospitalization ranged from -0.3593 to 0.8409.

We found that the lymphocyte count significantly increased between the first and seventh days of hospitalization (*p*=0.0089). Moreover, a proportional increase was observed in the levels of the CD4+ and CD8+ lymphocyte subpopulations (*p*=0.0052 and *p*=0.0065, respectively; Figure 4).

A non-significant increase in albumin levels was observed during hospitalization (*p*=0.8412). In addition, an important reduction was found in the mean PCR levels between the first and seventh days of hospitalization (144 mg/dL and 86 mg/dL, respectively); however, this reduction was not significant (*p*=0.0623).

## DISCUSSION

The mortality rate of our sample was 14.2%, which is within the parameters described for clinical and tertiary surgical care level I PICUs in developing countries 34.

On PICU admission, 33.3% of children were classified as malnourished; thus, the weight of these children was below the 10^th^ percentile of the W/A curve. This prevalence of malnutrition was similar to that found in other studies 1,2.

The acute nutritional impairment that results from the current acute illness was one of the possible causes for the high prevalence of low W/A because SIRS induces catabolism, and patients with sepsis have reduced food intake. Furthermore, the majority of patients had a chronic disease, which is common among children and adolescents admitted to a tertiary ICU.

We observed that 42.8% of the children were below the 3^rd^ percentile of the H/A curve (WHO, 2006), which suggests a chronic pattern of malnutrition. Two possibilities explain these findings: First, our service is a tertiary hospital, and numerous patients admitted to our units suffer from chronic diseases that can lead to chronic malnutrition. Second, chronically malnourished patients, even those without other diseases, are more prone to suffer from severe acute infections because their compromised immune systems lack macro- and micronutrients. These patients are also prone to immune hypo-responsiveness induced by increased IL-10 levels 7,35,36.

Upon admission, no patient was classified as overweight or at risk for overweight. On the 7^th^ day of hospitalization, two patients were considered overweight according to the WHO BMI curve. However, these weights were overestimated due to clinically evident anasarca that might be a consequence of hypoalbuminemia.

If we had considered hypoalbuminemia as a sign of malnutrition, then the prevalence of malnutrition in our sample would have risen from 33.3% to 100% since all the patients in our sample had albumin levels below the reference value. The deficiency of this protein contributed to third space fluid retention.

Another sign of nutritional impairment in our patients was anemia. Of the patients with anemia at ICU admission, 22% had hemoglobin levels below 7 g/dL, indicating a severe impairment.

Of all the micronutrients analyzed, we measured the zinc level because of its importance in lymphocyte development. The high prevalence of zinc deficiency in this population further corroborates their nutritional deficiencies. Another explanation for the low serum zinc levels associated with sepsis is the recruitment of zinc during the proliferation of immune cells and the formation of inflammatory proteins 12,29,37.

The presence of severe lymphopenia in 35% of the patients upon admission to the ICU can also be explained by the fact that the initial response to infectious agents is composed primarily of neutrophils and macrophages. Severe lymphopenia leads to the preferential recruitment of these cells relative to lymphocytes, which leads to transient lymphopenia.

Of the two patients who died during our study, one underwent testing only on the first day of hospitalization, and the other was tested on two days. Both patients had severe lymphopenia at each time of testing, with commitment of both lymphocyte phenotypes (CD4+ and CD8+). All surviving patients had normalized lymphocyte counts on the seventh day. This finding may reflect the poor prognosis of patients with prolonged lymphopenia, which is a sign of immunoparalysis. Other studies have reported these findings 17,21,25,30,32.

We observed that malnourished patients tended to have lower levels of CD8+ lymphocytes and higher levels of CD4+ lymphocytes on the seventh day of hospitalization than well-nourished children. However, this difference was not significant, possibly because of the small number of patients in our sample. No description of a predominant commitment of CD4+ or CD8+ T cells exists for patients with immunoparalysis. Rather, evidence shows the expansion of regulatory T cell (Treg) numbers (18,20,39). However, in this study, we did not evaluate Treg markers.

One limitation of our study is that we did not have access to methods, such as bioimpedance, to evaluate nutritional status and body composition. Furthermore, classic anthropometric measurements might be impaired among patients with liquid retention due to hypoalbuminemia, which is common in the PICU. For example, all of the patients in our sample had hypoalbuminemia upon admission. We also did not find correlations between the anthropometric parameters and either lymphocyte count or the presence of lymphopenia.

The concomitant increase in lymphocyte counts and zinc levels and the decrease in PCR levels reveals the important role that this micronutrient plays in lymphocyte development; furthermore, this relationship demonstrates its role in the inflammatory response, as other studies have shown 10,32,33,37. We suggest that other studies with more patients seek to verify the possible correlations among lymphocyte counts, zinc levels and PCR levels.

We did not find a correlation between nutritional status, including anthropometric measurements, and lymphocyte counts among patients admitted to the PICU. However, we did observe that malnourished patients tended toward lower CD8+ lymphocyte levels and higher CD4+ levels during the seventh day of hospitalization compared to those of well-nourished children. We also observed concomitant elevations of zinc levels and lymphocyte counts, which highlights the need to monitor micronutrient levels during PICU hospitalization to identify patients at risk for developing immunoparalysis.

More research is needed to identify additional parameters correlated with immunoparalysis to evaluate nutritional status and further elucidate the potential benefits of micronutrient supplementation among patients with sepsis.

## AUTHOR CONTRIBUTIONS

Manzoli TF was responsible for the data analysis and manuscript composition. Delgado AF was responsible for the study design, data analysis and manuscript revision. Troster EJ and de Carvalho WB were responsible for the manuscript revision. Antunes AC, Marques DM and Zamberlan P were responsible for the data acquisition.
